# Longitudinal Modes along Thin Piezoelectric Waveguides for Liquid Sensing Applications

**DOI:** 10.3390/s150612841

**Published:** 2015-06-02

**Authors:** Cinzia Caliendo

**Affiliations:** Istituto di Acustica e Sensori O. M. Corbino, IDASC-CNR, Via del Fosso del Cavaliere 100, 00133 Roma, Italy; E-Mail: cinzia.caliendo@idasc.cnr.it; Tel.: +39-6-4993-4741 or +39-6-4993-5439; Fax: +39-6-4548-8061

**Keywords:** BN, AlN, GaN, InN, ZnO, high frequency, liquid environment, coupling configurations, sensors

## Abstract

The propagation of longitudinally polarized acoustic modes along thin piezoelectric plates (BN, ZnO, InN, AlN and GaN) is theoretically studied, aiming at the design of high frequency electroacoustic devices suitable for work in liquid environments. The investigation of the acoustic field profile across the plate revealed the presence of longitudinally polarized Lamb modes, travelling at velocities close to that of the longitudinal bulk acoustic wave propagating in the same direction. Such waves are suitable for the implementation of high-frequency, low-loss electroacoustic devices operating in liquid environments. The time-averaged power flow density, the phase velocity and the electroacoustic coupling coefficient K^2^ dispersion curves were studied, for the first (S_0_) and four higher order (S_1_, S_2_, S_3_, S_4_) symmetrical modes for different electrical boundary conditions. Two electroacoustic coupling configurations were investigated, based on interdigitated transducers, with or without a metal floating electrode at the opposite plate surface. Enhanced performances, such as a K^2^ as high as 8.5% and a phase velocity as high as 16,700 m/s, were demostrated for the ZnO- and BN-based waveguides, as an example. The relative velocity changes, and the inertial and viscous sensitivities of the first symmetric and anti-symmetric mode, S_0_ and A_0_, propagating along thin plates bordered by a viscous liquid were derived using the perturbation approach. The present study highlights the feasibility of the piezoelectric waveguides to the development of high-frequency, integrated-circuits compatible electroacoustic devices suitable for working in liquid environment.

## 1. Introduction

The medical, food, and manufacturing industry, to name just a few, are increasingly demanding sensors for the investigation of liquids’ properties, such as viscosity, liquid identification and bio-sensing. Ultrasound sensors are made up of a piezoelectric waveguide that contacts the liquid environment: the sensing function is achieved through the perturbation of the acoustic wave (AW) velocity and attenuation by the electrical, mechanical or mass properties of the liquid. The quasistatic field associated with the AW is perturbed by either the conductivity or permittivity of the liquid contacting the sensor surface; small surface mass density changes at the sensor surface perturb the wave velocity; the AW propagation loss and velocity are affected by the liquid density and viscosity. A major issue of electroacoustic sensors for liquid detection is the polarization or the velocity of the AW that propagates along the waveguide. Waves with particle displacement components normal to the plate surface are highly lossy as they are converted into compressive waves in the surrounding liquid. This attenuation can be drastically reduced by exploiting acoustic modes travelling at velocities lower than the liquid compressional velocity. The fundamental antisymmetric Lamb mode, A_0_, can be designed to travel at a velocity lower than that of most liquids, which lie in the range from 900 to about 1500 m/s, by designing the proper acoustic waveguide thickness [1]. On the contrary, electroacoustic devices based on the propagation of *in-plane polarized* waves (such as shear horizontal acoustic plate modes (SHAPMs) or shear horizontal surface acoustic waves (SHSAW), or leaky longitudinal SAW (LLSAW)) dissipate energy into the liquid primarily by viscous coupling, and these energy losses are less severe than those suffered by devices based on the propagation of *elliptically polarized* SAWs [2,3,4]. The fundamental symmetric Lamb mode, S_0_, while propagating at phase velocity much higher than that of most liquids, with three particle displacement components (longitudinal, shear vertical and shear horizontal, hereafter named U_1_, U_2_ and U_3_) or two (U_1_ and U_3_), can show propagation conditions where the U_3_ component vanishes, making it suitable for operation in liquid environments. Such conditions are satisfied by the so called Anisimkin Jr. modes (AMs) [5,6]. The AMs propagate along thin plates at velocities close to that of the longitudinal bulk acoustic wave (LBAW), and are longitudinally polarized. The longitudinal displacement component is uniform through the plate depth (U_1_ = 1), being the U_2_ and U_3_ components negligible (U_2_ and U_3_ < 10%·U_1_) both at the plate surfaces and in the bulk. These modes are excited and detected by use of interdigitated transducers (IDTs), as for SAWs, but compared to the latter, they offer the advantage of an high velocity (hence higher operation frequency); moreover the liquid flow cell can be placed indifferently onto one of the plate surfaces, while the active area of the SAW-based sensors is only the region between the two IDTs.

A novel and exciting advancement in the development of AW sensors for liquids include both new viable modes and the selection of new materials, to design and fabricate fully portable, stand-alone AW sensor systems. Thus sensors are required showing a high electroacoustic coupling constant K^2^ (through the selection of appropriate coupling configurations), high operation frequency (through the use of proper piezoelectric materials showing a high phase velocity), high sensitivity, and optimized performances of the oscillator circuit. A sensor based on a high-frequency electroacoustic device exhibits superior performances: the higher the frequency the higher the sensor sensitivity and resolution. High-sensitivity viscosity sensors were implemented on *conventional* piezoelectric materials such as quartz, LiNbO_3_ and LiTaO_3_ [7] that unfortunately suffer from several limitations the most important are the limited acoustic velocity and the incompatibility with the Integrated Circuit (IC) technology. The so-called thin piezoelectric film technology overcomes these problems allowing *thin suspended membrane* structures to reach operation frequency in the high frequency band and is compatible with the mass electronic production IC technology. The dispersive behavior of the composite structures can be used to modulate the electroacoustic coupling efficiency, the velocity, the thermal behavior and the acoustic field profile of the propagating mode in order to achieve useful device functionalities.

Group-III nitrides, such as AlN, GaN, InN, and BN, and II-VI materials, such as ZnO, are the current focus of the present investigation since they possess a number of attractive properties that allow to fabricate novel device structures with great potential in high-power electronics and SAW devices applications. These piezoelectric thin films materials can be grown on silicon substrates by a variety of thin film deposition processes including sputtering, chemical vapor deposition or molecular beam epitaxy, with or without the presence of a seed layer to reduce the lattice mismatch between the film and the substrate. These films can crystallize in a hexagonal wurtzite structure with epitaxial structural quality up to a few micrometers thickness. Despite the fact ZnO exhibits low acoustic velocity, high acoustic loss and a large temperature coefficient of frequency (TCF) that limit its applications in high-frequency electroacoustic devices, it has the largest electroacoustic coupling efficiency, K^2^, among the studied materials. GaN is a very hard, mechanically stable, wide bandgap semiconductor material with high heat capacity and thermal conductivity. GaN is a key material for the next generation of high frequency and high power transistors; is considered one of the most important semiconductors after Si. Among all, AlN is a highly attractive thin film material for applications in electroacoustic devices because of its high acoustic velocity, good thermal conductivity, moderate piezoelectric coupling coefficient, low acoustic loss, and relatively small TCF. BN has excellent properties, such as stability at high temperatures, extreme hardness, only second to diamond, and a low chemical reactivity, large band gap, but quite low K^2^. wz-BN films are currently grown by reactive sputtering technique on Si for high frequency SAW devices [8,9]. In combination with AlN, the BN/AlN-based composite plates [10] allow the propagation of fast longitudinal modes with high K^2^. InN is perhaps the less studied material among all even if its applications to SAW devices and sensors were predicted since 1998 [11]. c-axis oriented InN films have been deposited by reactive RF magnetron sputtering both on bare and Pt coated Si(100) substrates, and on Si(100) by RF metal organic molecular beam epitaxy system [12,13,14].

In the present paper the theoretical performances of BN, AlN, GaN, InN and ZnO-based electroacoustic devices have been explored for liquid sensing applications where the AMs-based device acts as a readout technique. Such sensors offer the advantage to work at high frequency, to be fully compatible with the IC technology and suitable for prospective applications in remote acoustic sensing.

## 2. Theoretical Analysis

**Figure 1:**
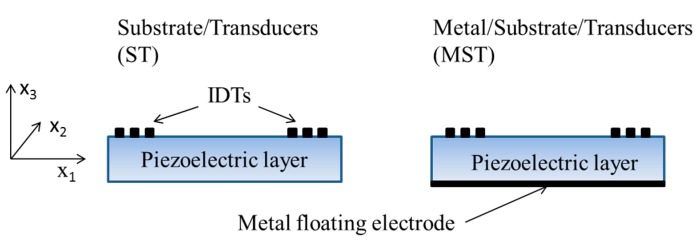
Cross sections of the Substrate/Transducer (ST) and Metal/Substrate/Transducer (MST) coupling configurations.

The phase velocity dispersion curves of the A_0_, S_0_ and higher order symmetric Lamb modes propagating along thin anisotropic plates of finite thickness has been calculated for different piezoelectric materials (BN, AlN, GaN, InN, ZnO), thicknesses and electrical boundary conditions. The calculations were performed by using Matlab routine in the lossless approximation; the material data (mass density, elastic, piezoelectric, and dielectric constants) are referred to [15,16]. By considering the electrically opened and shorted boundary conditions at the plate surfaces, the effect of a thin massless metallization was simulated that results in the two different coupling structures shown in Figure 1, with the IDTs placed on one of the plate surfaces, with (Metal/Substrate/Transducer, MST) or without (Substrate/Transducer, ST) a floating electrode on the opposite one. Here the mechanical effect of the metallization is ignored as the metallization is assumed to be infinitely thin.

The dispersion curves of the phase and group velocity, *v*_ph_ and $v_{g}=v_{ph}^{2}/(v_{ph}-\left(fh\right)\frac{dv_{ph}}{d\left(fh\right)})$, of the fundamental and higher order modes were calculated for each material, where *h* is the plate thickness and *f* the frequency. The K^2^ dispersion curves were calculated according to the approximated formula $K^{2}=2\cdot \left(v_{ph}^{f}-v_{ph}^{m}\right)/v_{ph}^{f}$, where $v_{ph}^{m}$ and $v_{ph}^{f}\;$ are the phase velocities along the plate, with and without a metal floating electrode in the IDT region, in the approximation of infinitesimally-thin perfectly-conductive floating electrodes. The K^2^ *vs.* *h*/λ curves were studied for the two coupling configurations and for each piezoelectric material, being λ the acoustic wavelength. The power transported by the Lamb mode along the plate per unit length perpendicular to the propagation direction and per unit time, $P_{n}=\frac{1}{2}\text{ρ}{\text{ω}}^{2}v_{gr}^{n}\int_{-h/2}^{h/2}\left({\left|u_{1}\right|}^{2}+u_{3}{}^{2}\right)dx_{3}$, was calculated for waves propagating along the *x*_1_ direction of the plate, being *u_1_* and *u_3_* the longitudinal and transverse particle displacement components normalized to the power flow evaluated at the *x*_3_ = *h*/2 surface; ω *=* 2π*·f* is the angular frequency, *f = v_ph_/*λ, ρ the plate mass density, and *n* is the mode order. The power flow density *P_n_* distribution inside the plate gives information about the location of the peaks of acoustic energy transmission [17]. The power flow analysis was performed for a few higher order symmetric modes travelling at the LBAW velocity.

## 3. Acoustic Wave Propagation along Thin Waveguides

The piezoelectric acoustic waveguide theoretically investigated here can be fabricated by using conventional thin film deposition techniques and bulk micromachining of (001)Si to obtain a thin suspended piezoelectric membrane. The fabrication procedure of the acoustic waveguide is compatible with semiconductor processing techniques, thus offering the advantage of providing the monolithic integration of the device with the signal processing electronics. The wurtzite piezoelectric materials studied here (AlN, BN, InN, GaN, ZnO) are isotropic in the c-plane, and hence the velocity of the surface and bulk acoustic waves is unaffected by the propagation direction selected in this plane. The SAW propagating along the (001) plane is a Rayleigh-type wave that has only two non-null rapidly damped displacement components, U_1_ and U_3_, lying in the sagittal plane. The electroacoustic coupling coefficient K^2^ of the Rayleigh-like waves, as well as the velocities of the SAW and BAWs propagating along the <100> direction of the (001) plane are listed in Table 1.

**Table 1:** The K^2^ and the velocity of the Rayleigh-like SAW, the velocities of the longitudinal (LBAW), shear vertical (SVBAW) and shear horizontal (SHBAW) BAWs propagating along the <100> direction of AlN, GaN, InN, ZnO and BN crystals.

Material	K^2^ (%)	*v_SAW_* (m/s)	*v_LBAW_* (m/s)	*v_SHBAW_* (m/s)	*v_SVBAW_* (m/s)
AlN	0.28	5607	10,287	5814	6089
GaN	0.16	3701	7756	3861	4277
InN	0.61	2608	5722	2816	2731
ZnO	1.0	2681	6074	2804	2829
BN	0.04	9719	16,781	11,027	10,587

**Figure 2:**
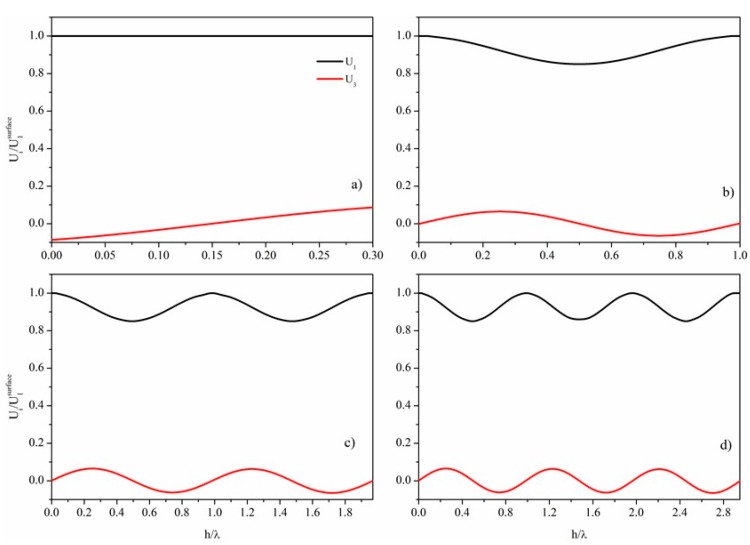
Cross sectional normalized distribution of normal (U_3_) and longitudinal (U_1_) displacements for: (**a**) AM propagating along the BN plate with h_BN_/λ = 0.3; (**b**) S_1_ mode for h_BN_/λ = 1; (**c**) S_2_ mode for h_BN_/λ = 1.97; (**d**) S_3_ mode for h_BN_/λ = 2.95.

The fundamental *symmetric* Lamb mode, S_0_, as well as the higher order modes, are elliptically polarized and have higher velocity than the surrounding liquid medium: thus they are not suitable for sensing applications in liquids except in some special cases. These cases include: (1) the linearly polarized AMs that are a branch of the S_0_ mode; (2) the higher order *symmetric* modes just for specific plate normalized thicknesses. The former propagate at a velocity a little lower than the *v_LBAW_*, while the latter at the velocity of the LBAW. Unlike the AMs, for these modes the displacement component U_3_ vanishes on the plate surfaces, while it shows non-null values into the bulk of the plate, as shown in Figure 2 where the field profile of the fundamental and of the three higher order symmetric modes (S_1_, S_2_ and S_3_) propagating along a BN plate are depicted. Here the BN field profile has been taken as example of the behavior of all the other piezoelectric materials. Figure 2a shows the acoustic field profile of the AM propagating along a BN plate 0.3λ thick. Figure 2b–d show the acoustic field profile of the S_1_, S_2_ and S_3_ modes propagating along the BN plate with normalized thickness h_BN_/λ =1, 1.97, and 2.95, respectively. The displacement amplitudes are normalized by the U_1_ value at the free surface of the plate.

As it can be seen, the through-thickness for U_1_ and U_3_ are symmetric and antisymmetric about the mid plane of the plate, respectively, and the number of the minima increases with increasing the mode order. As the U_3_ component of these higher order modes vanishes on the free surfaces of the plate and the U_1_ component dominates across the entire thickness, these modes are suitable for liquid sensing applications as well as the AMs.

**Figure 3:**
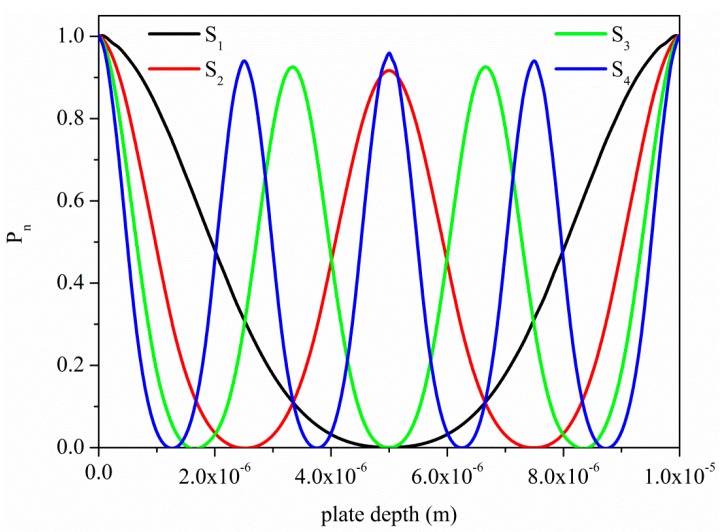
Normalized time-averaged power flow density P_n_ of the first four symmetric modes propagating along a ZnO plate 10 µm thick.

Figure 3 shows the time-averaged power flow density of the first four higher order modes, S_1_, S_2_, S_3_ and S_4_, propagating along a ZnO plate, normalized to the surface value. As it can be seen in Figure 3, with increasing the mode order *n*, more energy is concentrated near the free surfaces of the plate [17]; the higher the order, the higher the operation frequency, at the same plate thickness. Here the ZnO plate has been taken as example of the behavior of all the other piezoelectric materials; a thickness of 10 µm is the typical threshold value for a sputtered piezoelectric layer still showing a fairly good c-axis orientation. All these modes propagate at the same velocity along the ZnO plate, 10 µm thick, with different wavelengths λ_n_ (and hence frequency$\;f_{n}=v_{ph}/\lambda_{n}$), imposed by the corresponding normalized thickness value.

### 3.1. Acoustic Waves Propagation along BN Thin Plates

**Figure 4:**
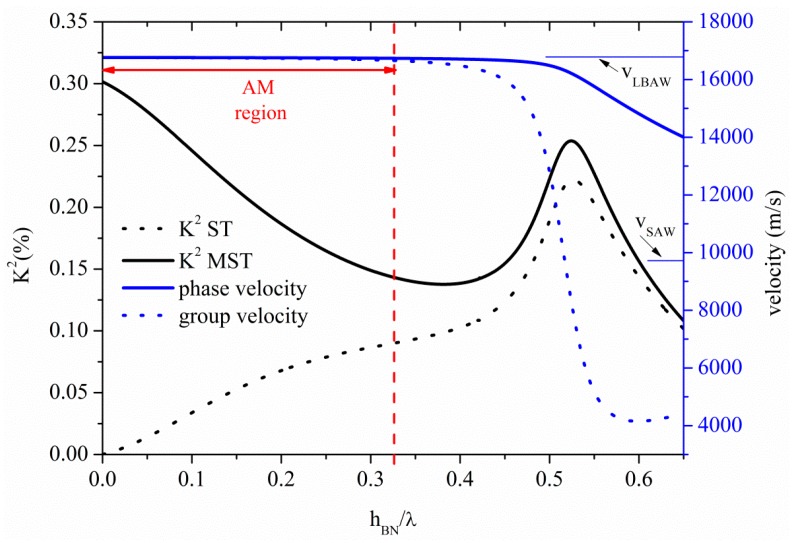
Phase and group velocity, and K^2^ dispersion curves of the S_0_ mode propagating along the MST and ST configurations on BN plate.

The phase and group velocity dispersion curves of the S_0_ mode propagating along a BN plate are characterized by a low dispersion region, where the two velocities coincide, are almost constant and quite close to the *v_LBAW_*, as shown in Figure 4. Both the phase and group velocities of Figure 4 are referred to the propagation along the bare BN plate.

The abscissa of Figure 4 represents the BN thickness-to-wavelength ratio, h_BN_/λ. In the flat phase velocity dispersion region the U_1_ component of the S_0_ mode dominates over the U_3_, being U_2_ = 0. The threshold BN plate thickness after which the AMs no longer propagates is h_BN_/λ = 0.325, as shown in Figure 4 where the AM region is pointed out by a double-headed arrow. For h_BN_/λ ≤ 0.325, the mode propagate at velocity of 16,735 m/s, the U_1_ component is uniform along the plate depth and the U_3_ component is less than 10% of U_1_. For h_BN_/λ > 0.325, the mode transforms to the quasi longitudinal mode (QLM): U_1_ is no longer constant inside the plate (but still dominant over the other components), U_2_ = 0 and U_3_ > 10%·U_1_. With increasing h_BN_/λ, U_3_ increases and the mode polarization becomes elliptical. When the mode velocity approaches the shear horizontal BAW velocity, the U_2_ component largely dominates over U_1_ and U_3_. For h_BN_/λ > 1, the mode begins to act as a Rayleigh wave propagating on the surfaces of the plate.

The K^2^ dispersion curves of the S_0_ mode for the two transducers configurations, are also shown in Figure 4: despite the remarkable high phase velocity, the BN piezoelectricity is unfortunately weak, and thus its K^2^ is quite low. Given the small thickness of the studied S_0_-based plates, the IDTs fingers are quite close to the opposite metal floating electrode for the MST configuration and consequently the electric field is mainly perpendicular to the plate surfaces. This results in a coupling efficiency higher than that obtainable for the ST configuration, when the mode is longitudinal. The K^2^ of the MST configuration is quite larger than that of the ST for h_BN_/λ < 0.3, where the longitudinally polarized mode is electrically coupled through the piezoelectric strain constant d_31_. The floating electrode magnifies the IDTs crossed electric field component that is dominant over the in-line one thus making more efficient the electroacoustic transduction for the S_0_ mode-based MST configuration. With increasing the plate thickness, the K^2^ of the two structures merge. The K^2^ is thus determined according to which direction of the electric field the piezoelectricity of the plate is most sensitive, being the sensitivity thickness-dependent.

The S_1_, S_2_ and S_3_ higher order symmetric modes propagate along the BN plate at *v_LBAW_* for h_BN_/λ = 1, 1.97, and 2.95; their K^2^ is 0.035% (0.04%), 0.018% (0.020%), 0.012% (0.013%), for the ST (MST) configuration, quite lower than that of the AMs. As it can be seen in Figure 4, the K^2^ of the AM ranges from 0.3% to 0.14% and from 0% to 0.09%, when considering the ST and MST configurations, respectively. For a plate with a fixed thickness of 10 µm, the operating frequencies of the S_1_, S_2_ and S_3_ modes: 1.7, 3.3, and 5.0 GHz, respectively, are much higher than that (0.5 GHz) of the AM calculated for h_BN_/λ = 0.325, because of their different λ. Unlike the AM mode, where phase and group velocities are very close, higher order modes indeed are characterized by a high phase velocity dispersion.

**Figure 5:**
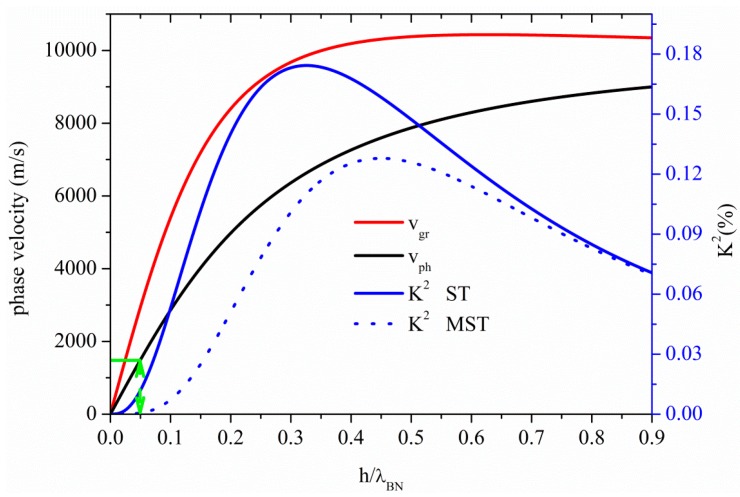
Phase and group velocity, and K^2^ dispersion curves of the A_0_ mode propagating along the MST and ST configurations on BN plate.

Figure 5 shows the phase and group velocity, and the K^2^ dispersion curves of the A_0_ mode propagating along the BN plate, for the two coupling structures. At very small thickness-to-wavelength ratios, the phase velocity of the A_0_ mode approaches zero; as h_BN_/λ increases, the velocity also increases, and reaches asymptotically from below the BN SAW velocity. The particle motion of the A_0_ mode is predominantly normal to the plate for very thin plates. Unlike the S_0_ mode, for the A_0_ one the ST configuration shows a K^2^ higher than that of the MST. At very small h_BN_/λ, the mode polarization is predominantly shear vertical (U_3_) and the mode is electrically coupled through the piezoelectric strain constant d_15_, generating the shear strain S_13_. The transduction coupling is produced by the in-line component of the IDTs electric field that is dominant in absence of the floating electrode. The horizontal green line in Figure 5, representing the water wave velocity (1480 m/s), crosses the phase velocity curve at h/λ = 0.05, that represents the cut-off condition for operation in water. The corresponding K^2^ values as low as 0.011% and 0% for the ST and MST structures, respectively, suggests that the A_0_ mode is impractical for liquid sensing.

The results shown in Figure 4 and Figure 5 indicate that the S_0_ mode usually exhibits a K^2^ larger than that of the A_0_ mode for both the two configurations. Moreover the metallized bottom surface enhances the K^2^ of the S_0_ mode and reduces that of the A_0_ mode. The conductive layer on the backside of the BN thin film has the double role to boost the K^2^ of the S_0_ mode and to reduce the coupling strength of the unwanted A_0_ mode that appears as a spurious mode in the frequency spectrum. This effect is visible in all the materials analyzed in the present paper.

### 3.2. Acoustic Waves Propagation along ZnO Thin Plates

**Figure 6:**
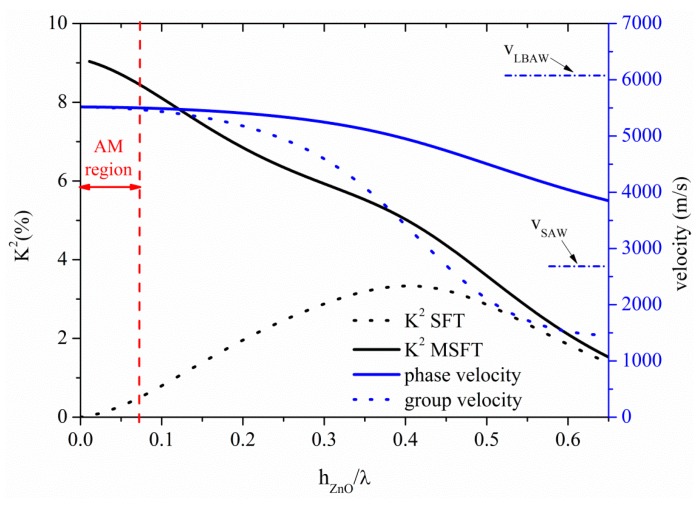
Phase and group velocity, and K^2^ dispersion curves of the S_0_ mode propagating along the MST and ST configurations on ZnO plate.

Figure 6 shows the K^2^, phase and group velocity dispersion curves of the S_0_ mode propagating along the ZnO plate, for the ST and MST configurations. For very thin plates, the S_0_ mode propagates at a velocity *v* = 5500 m/s, quite lower than the LBAW velocity. The threshold ZnO plate thickness after which the AMs no longer propagates is h_ZnO_/λ = 0.07; the K^2^ ranges from 9% to 8.5% and from 0% to 0.47% along the AM region indicated by a double-headed arrow in Figure 6, for the MST and ST configurations. Among all the AMs-based piezoelectric plates here studied, the ZnO-based MST configuration is the one that offers the highest K^2^ value, followed by the InN-, AlN-, GaN- and BN-based ones.

Higher order symmetric modes S_1_, S_2_ S_3_, and S_4_, propagate at *v_LBAW_* for h_ZnO_/λ ~ 0.65, 1.24, 1.86, 2.48. Their K^2^ is equal to 0.42% (0.50%), 0.22% (0.24%), 0.15% (0.16%), and 0.10% (0.13%), respectively referred to the ST (MST) configurations, quite lower than that of the AMs. For 10 µm thick ZnO membrane, the S_1_, S_2_, S_3_ and S_4_-based devices operating frequencies are 394 MHz, 754 MHz and 1125 MHz and 1507 MHz, respectively, as opposed to that (38 MHz) estimated at the upper limit of the AM region.

**Figure 7:**
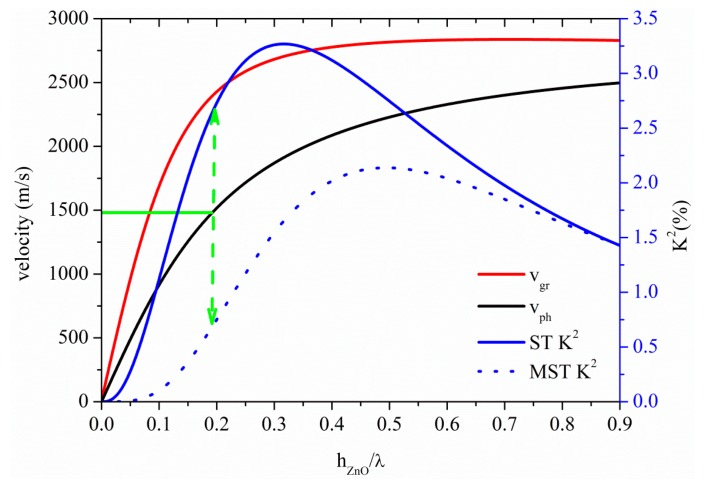
Phase and group velocity, and K^2^ dispersion curves of the A_0_ mode propagating along the MST and ST configurations on ZnO plate.

Figure 7 shows the phase and group velocity, and the K^2^ dispersion curves of the A_0_ mode propagating along the ZnO plate, for the two coupling structures. At the cut-off condition for operation in water (h_ZnO_/λ = 0.19), K^2^ is equal to 2.63% and 0.7% for the ST and MST structures, as shown by the dotted green arrow. Among all the A_0_ based piezoelectric plates here studied, the ZnO-based ST configuration is the one that has the highest K^2^ together with the largest plate thickness value corresponding to *v_ph_* ≤ *v_water_* = 1480 m/s , followed by the InN-, GaN-, AlN- and BN-based ones. As for InN, the low SAW velocity of the ZnO allows the A_0_ mode to be suitable for liquid sensing applications at plate thickness values higher than those relative to faster SAW materials.

### 3.3. Acoustic Waves Propagation along AlN Thin Plates

**Figure 8:**
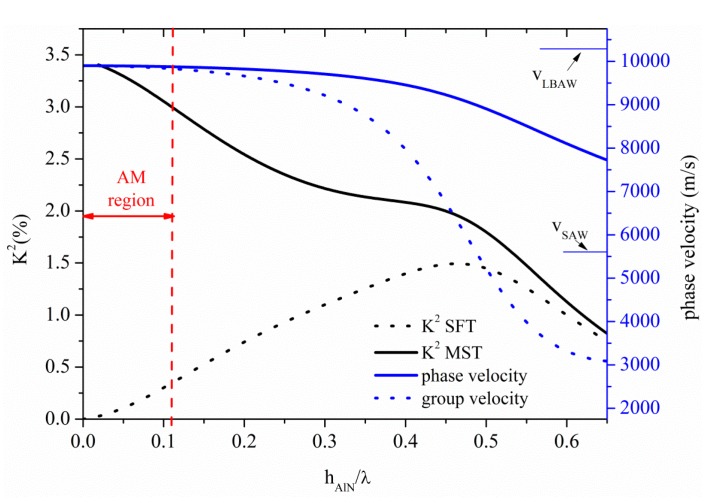
Phase and group velocity, and K^2^ dispersion curves of the S_0_ mode propagating along the MST and ST configurations on AlN plate.

The S_0_ Lamb mode along AlN thin plate propagates at velocity quite lower than the LBAW velocity, as shown in Figure 8 where the S_0_ velocity and K^2^ dispersion curves are shown for the ST and MST configurations. The threshold AlN plate thickness after which the AMs no longer propagates is 0.11, with K^2^ ranging from 3.4% to 3% and from 0% to 0.35% along the AM region indicated by a double-headed arrow in Figure 8, for the MST and ST configurations. The S_0_ mode in AlN plate is attractive because it combines high velocity (9875 m/s), low dispersive velocity characteristic, and a quite good K^2^.

Higher order modes, corresponding to the S_1_, S_2_, S_3_ and S_4_, can be found, for example, at h_AlN_/λ ~ 0.79, 1.58, 2.37 and 3.16, travelling at LBAW velocity and showing K^2^ = 0.31% (0.37%), 0.17% (0.19%), 0.11% (0.13%), and 0.08% (0.10%) for ST (MST) configurations. The corresponding AM and higher order modes operating frequencies, for the same plate thickness (h=10 µm) are 106 MHz, 790 MHz, 1.62 GHz, 2.4 GHz, and 3.25 GHz, respectively.

**Figure 9:**
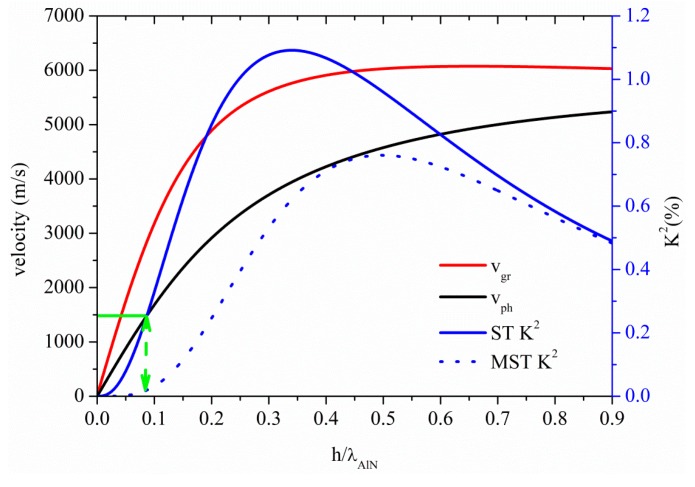
Phase velocity and K^2^ dispersion curves of the A_0_ mode propagating along the MST and ST configurations.

Figure 9 shows the phase and group velocity, and the K^2^ dispersion curves of the A_0_ mode propagating along the AlN plate, for the two coupling structures. The horizontal green line crosses the A_0_ mode velocity curve at 1480 m/s, the water velocity value: the corresponding K^2^ values are 0.25% and 0.02% for the ST and MST structures, for h_AlN_/λ = 0.087, as shown by the dotted green arrow.

### 3.4. Acoustic Waves Propagation along GaN Thin Plates

**Figure 10:**
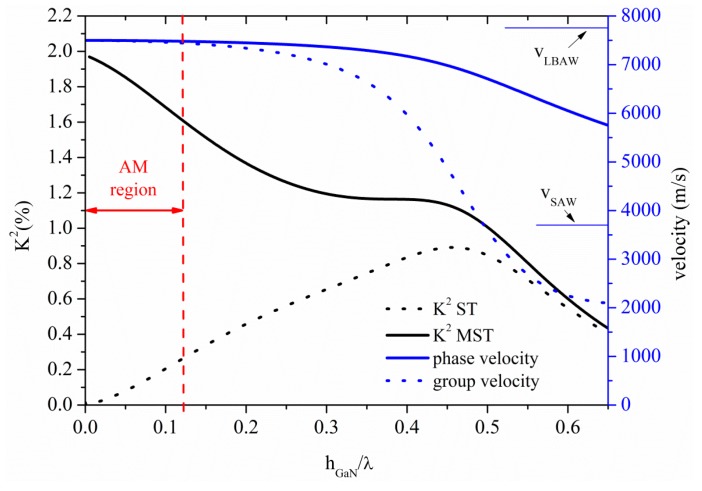
Phase velocity and K^2^ dispersion curves of the S_0_ mode propagating along the MST and ST configurations on GaN plate.

The S_0_ Lamb mode along GaN thin plate propagates at velocity quite lower than *v_LBAW_*, as shown in Figure 10 where the S_0_ velocity and K^2^ dispersion curves are shown for the ST and MST configurations. The threshold GaN plate thickness after which the AMs no longer propagates is h_GaN_/λ = 0.12, at velocity 7479 m/s; K^2^ ranges from 2% to 1.61% and from 0% to 0.26% along the AM region indicated by a double-headed arrow in Figure 10, for the MST and ST configurations.

The first four higher order symmetric modes propagate at *v_LBAW_* along the GaN plate for h_GaN_/λ = 0.77, 1.53, 2.29 and 3.05, respectively, and with K^2^ equal to 0.18% (0.20%), 0.095% (0.1%), 0.06% (0.07%), 0.05% (0.05%) for the ST (MST) configurations. For 10 µm thick GaN membrane, the operating frequencies of the S_1_, S_2_, S_3_ and S_4_-based devices are 597, 1193, 1939 and 2585 MHz, respectively, as opposed to that (90 MHz) estimated at the upper limit of the AM region.

**Figure 11:**
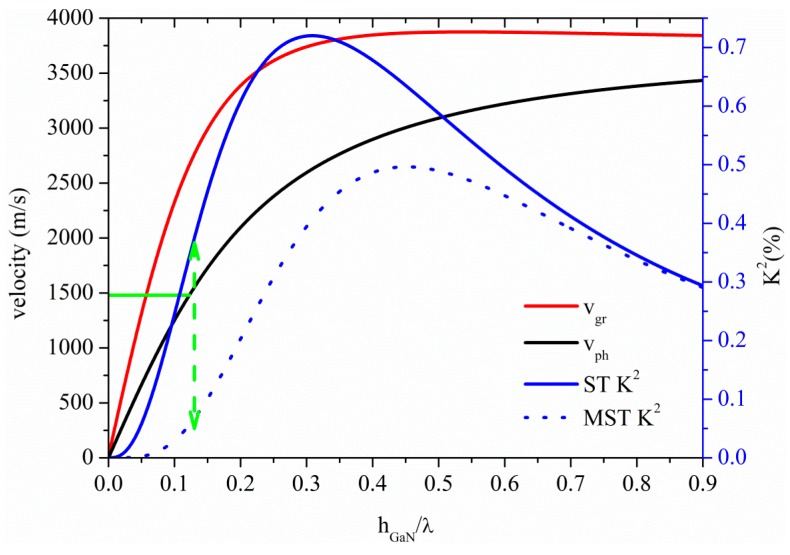
Phase and group velocity, and K^2^ dispersion curves of the A_0_ mode propagating along the MST and ST configurations on GaN plate.

The phase and group velocity, and K^2^ dispersion curves of the A_0_ mode travelling along the GaN plate are shown in Figure 11, for the two coupling configurations. The horizontal green line crosses the A_0_ phase velocity curve at 1480 m/s for h/λ = 0.12: the corresponding K^2^ values are 0.35% and 0.06% for the ST and MST structures, as shown by the dotted green arrow.

### 3.5. Acoustic Waves Propagation along InN Thin Plates

**Figure 12:**
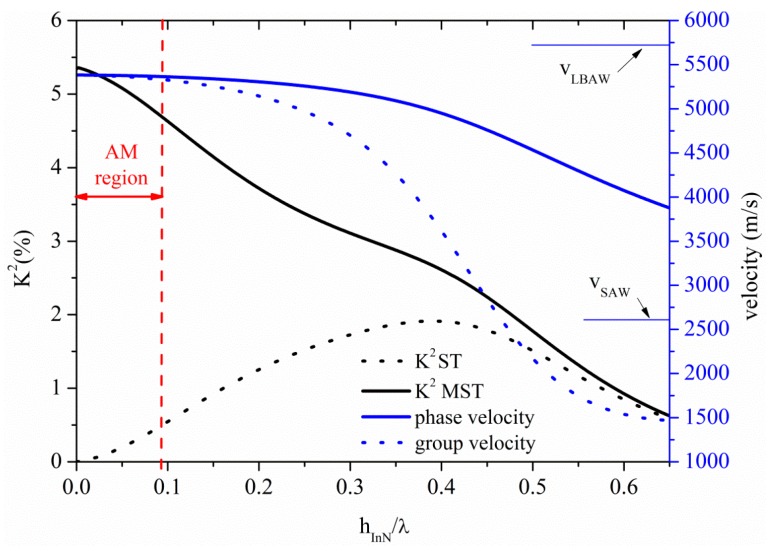
Phase and group velocity, and K^2^ dispersion curves of the S_0_ mode propagating along the MST and ST configurations on InN plate.

The S_0_ Lamb mode propagates along the InN plate at velocity lower than that of the LBAW as shown in Figure 12 where the velocity and K^2^ dispersion curves are shown for the ST and MST configurations. The threshold InN plate thickness after which the AMs no longer propagate is 0.09 at velocity 5365 m/s, with K^2^ ranging from 5.4% to 4.73% and from 0% to 0.5%, along the AM region indicated by a double-headed arrow in Figure 12, for the MST and ST configurations.

The higher order symmetric modes S_1_, S_2_, S_3_ and S_4_ propagate along InN plate at h_InN_/λ = 0.67, 1.33, 2.01 and 2.67, respectively; the K^2^ of these modes is equal to 0.43% (0.5%), 0.23% (0.25%), 0.15% (0.17%), 0.12% (0.13%), for the ST (MST) configuration, quite lower than that of the AM. For 10 µm thick InN membrane, the operating frequencies of the S_1_, S_2_, S_3_ and S_4_-based devices are 381 MHz, 762 MHz, 1144 MHz and 1546 MHz, respectively, as opposed to that (48 MHz) estimated at the upper limit of the AM region.

**Figure 13:**
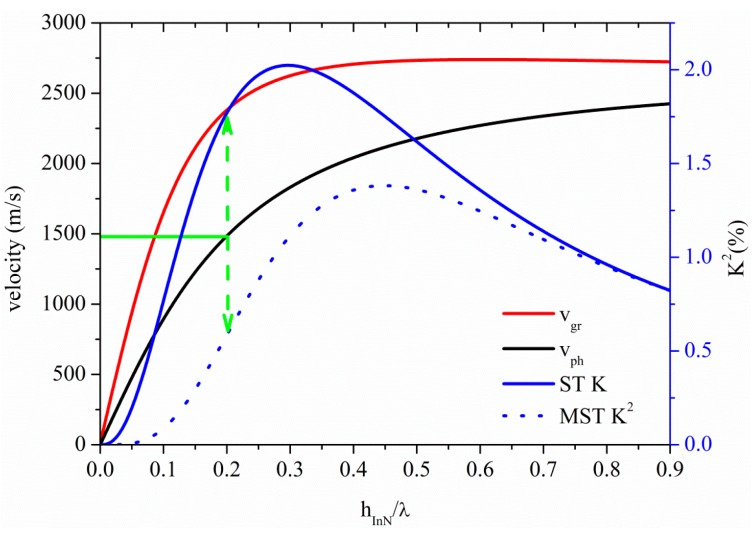
Phase and group velocity, and K^2^ dispersion curves of the A_0_ mode propagating along the MST and ST configurations on InN plate.

Figure 13 shows the phase and group velocity, and the K^2^ dispersion curves of the A_0_ mode propagating along the InN plate, for the two coupling structures. The horizontal green line crosses the A_0_ phase velocity curve at 1480 m/s at h_InN_/λ = 0.2, as shown by the horizontal green line: the corresponding K^2^ values are 1.77% and 0.58% for the ST and MST structures, as shown by the dotted green arrow. InN and ZnO-based A_0_ mode devices show the highest K^2^ for *v* ≤ 1480 m/s among the materials here studied.

## 4. Liquid Sensor

When a liquid contact the acoustic waveguide, the in-plane particle displacement component of the acoustic mode couples to a very thin viscous boundary layer, while the out-of-plane component couples to the inertia of the liquid layer. The AMs are a very attractive choice for sensing application in liquids as they have only one source of attenuation, the friction between the surface plate and the adjacent liquid. The systems studied (x_1_-propagation in the c-plane of the BN, AlN, ZnO, InN and GaN) are plates of thickness h, extending to infinity in both x_1_ and x_2_ directions. The perturbation method was used to calculate both the inertial and viscous relative velocity shifts, Δ*v/v_in_* and Δ*v/v_visc_*, that arise from the liquid mass and viscous loadings on the A_0_ and S_0_ modes propagating along the thin plates, for each piezoelectric material [18,19]. The calculations were performed under the hypothesis that the thin piezoelectric plate is symmetrically bordered on both surfaces by a layer of homogeneous liquid (water). The liquid layer can be considered as a small perturbation of the unperturbed plate provided that its acoustic impedance is much smaller than that of the plate, and it is greater than the viscous boundary layer thickness δ_η_ = [2η/ρ_1_ω]^1/2^, corresponding to the decay length for the longitudinal mode radiated into the liquid by the oscillating waveguide surface, being ρ_l_ and η the liquid mass density and shear viscosity.

**Figure 14:**
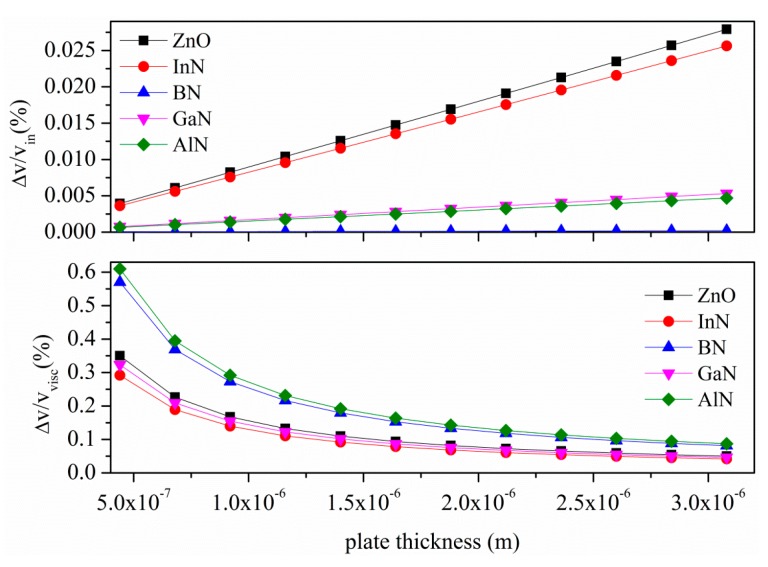
S_0_ mode relative velocity changes, Δ*v/v_in_* and Δ*v/v_visc_*, due to the inertial and viscous effects of the water contacting the plate, *vs.* the plate thickness.

Figure 14 shows the relative velocity shifts of the S_0_ mode due to the water inertial and viscous loading (assuming *v*_l_ = 1500 m/s, ρ_l_ = 1000 kg/m^3^ and η =10^−3^ Pa·s as the water velocity, mass density and viscosity). Assuming to operate at the same frequency of 65 MHz (δ_η_ = 7 × 10^−2^ µm) for all the cases under consideration, the corresponding wavelengths λ are: 75, 256, 75, 110, 148 µm, for ZnO, BN, InN, GaN and AlN, respectively. The plate thickness range (0.44 to 3.1 μm) considered in Figure 14 is within the AM range of existence.

**Figure 15:**
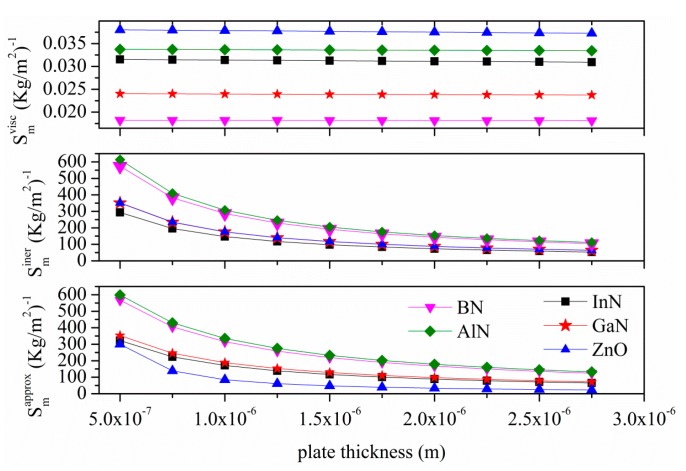
Inertial effect sensitivity ($S_{m}^{iner}$), viscous effect sensitivity ($S_{m}^{visc}$) and approximated sensitivity ($S_{m}^{approx}$) for the A_0_ mode *vs.* plate thickness due to the loading of a viscous liquid (water).

Figure 15 shows the sensitivities (to the inertial and viscous effects) of the A_0_ mode vs the plate thickness as derived by the perturbation theory other than the approximated gravimetric sensitivity $S_{m}^{approx}$ from [20]. An operating frequency of 4.7 MHz and a plate thickness range from 0.5 to 2.75 µm are assumed for each material. The approximated mass loading sensitivity $S_{m}^{approx}=\left(\Delta v/v\right)/m^{\prime }$, was calculated by the fractional velocity change $\text{Δ}v/v=1-{\left[1+\left({\text{ρ}}_{l}\cdot {\text{δ}}_{e}+{\text{ρ}}_{l}\cdot {\text{δ}}_{\eta }\right)/M\right]}^{-1/2}$, being ${\text{δ}}_{e}=\left(\text{λ}/2\text{π}\right){\left[1-{\left(v/v_{l}\right)}^{2}\right]}^{-1/2}\;$ the A_0_ mode evanescent decay length $m^{\prime }={\text{ρ}}_{l}\cdot {\text{δ}}_{e}+{\text{ρ}}_{l}\cdot {\text{δ}}_{\eta }$, $\rho_{l}\cdot \delta_{e}$ the equivalent mass loading of the liquid, ${\text{ρ}}_{l}\cdot {\text{δ}}_{\eta }$ the equivalent viscous loading of the liquid, and M = ρ_plate_·h the plate mass per unit width and length [20]. Assuming that $\left({\text{ρ}}_{l}\cdot {\text{δ}}_{e}+{\text{ρ}}_{l}\cdot {\text{δ}}_{\eta }\right)/M\ll 1$, the sensitivity may be approximated as $S_{m}^{approx}\approx 1/2\cdot M$.

## 5. Conclusions

This paper proposes the theoretical analysis of Lamb waves propagating along thin piezoelectric membranes with two types of electrode arrangement, aimed at the development of a small, completely integrated sensor for monitoring the properties of liquids. We report on the observation of very-high-velocity guided modes along thin plates with thickness about an order of magnitude smaller than the wavelength. The propagation characteristics of very fast longitudinal modes, the Anisimkin Jr. plate modes, along thin piezoelectric plates is studied for different piezoelectric materials (BN, AlN, GaN, InN, ZnO), their thickness, and the electrical boundary conditions. The velocity and the electroacoustic coupling efficiency K^2^ dispersion curves, as well as the acoustic field profile, and the time-averaged power flow density, were calculated for each material plate. The gravimetric sensitivity of the S_0_ and A_0_-mode based piezoelectric plates was theoretically predicted, specifically addressing the design of enhanced-coupling, microwave frequency mass sensors for applications in probing the solid/liquid interface. Provided that both the A_0_ and the AMs device function well in a liquid environment, they are good candidates for biosensing and chemical sensing in liquids. Because the thin suspended membrane of such modes device may be only a few micrometers thick, the mass per unit area of the thin plate can be increased significantly by the mass-loading effect produced either by the adsorption of chemical vapor molecules onto the plate, or by the changes in the density of a fluid on the plate, or by the attachment onto the plate of protein molecules, cells, and bacteria from a liquid that contact the plate. Moreover, it is worth noting that the large-amplitude motion of the modes studied can produce kinetic effects suitable for applications such as transport of granular solids or pumping and mixing of fluids. As a result, the Lamb modes device is able to work as a sensor as well as an actuator.

## References

[1] Caliendo C. (2013). Theoretical investigation of Lamb wave A_0_ mode in thin SiC/AlN membranes for sensing application in liquid media. Sens. Actuators B Chem..

[2] Wu J., Zhu Z. (1996). Sensitivity of Lamb Wave Sensors in Liquid Sensing. IEEE Trans. Ultrason. Ferroelectr. Freq. Control.

[3] Nayfeh A.H., Nagy P.B. (1997). Excess attenuation of leaky Lamb waves due to viscous fluid loading. J. Acoust. Soc. Am..

[4] Caliendo C., D’Amico A., Verardi P., Verona E. K+ detection using shear horizontal acoustic modes. Proceedings of the IEEE International Ultrasonics Symposium (IUS).

[5] Anisimkin V. (2010). General Properties of the Anisimkin Jr. Plate Modes. IEEE Trans. Ultrason. Ferroelectr. Freq. Control.

[6] Gulyaev Y.V. Propagation of the Anisimkin Jr.’ plate modes in LiNbO_3_ and Te single crystals. Proceedings of the 2008 IEEE International Ultrasonics Symposium (IUS).

[7] Anisimkin I.V., Anisimkin V.I. (2006). Attenuation of Acoustic Normal Modes in Piezoelectric Plates Loaded by Viscous Liquids. IEEE Trans. Ultrason. Ferroelectr. Freq. Control.

[8] Hu C., Kotake S., Suzuki Y., Senoo M. (2000). Boron nitride thin films synthesized by reactive sputtering. Vacuum.

[9] Sun L., Chen X. Research on the h-BN films for high frequency SAW devices. Proceedings of the SPIE 8202 International Conference on Optical Instruments and Technology: Solid State Lighting and Display Technologies, Holography, Speckle Pattern Interferometry, and Micro/Nano Manufacturing and Metrology.

[10] Caliendo C. (2015). Analytical Study of the Propagation of Fast Longitudinal Modes along wz-BN/AlN Thin Acoustic Waveguides. Sensors.

[11] Ambacher O. (1998). Growth and applications of Group III-nitrides. J. Phys. D Appl. Phys..

[12] Cao C.B., Chan H.L.W., Choy C.L. (2003). Piezoelectric coefficient of InN thin films prepared by magnetron. Thin Solid Films.

[13] Moret M., Ruffenach S., Briot O., Gil B., Pauthe M. (2009). The epitaxial growth of indium nitride using berlinite (AlPO_4_) and other piezoelectric crystals of the quartz family as substrates. Appl. Phys. Lett..

[14] Chen W.-C., Kuo S.-Y. (2012). Study of high quality Indium Nitride films grown on Si(100) substrate by RF-MOMBE with GZO and AlN buffer layer. J. Nanomater..

[15] Levinshtein M.E., Rumyantsev S.L., Shur M.S. (2008). Properties of Advanced Semiconductor Materials: GaN, AIN, InN, BN, SiC, SiGe.

[16] Choy M.M., Cook W.R., Hearmon R.E.S., Jaffe H., Jefphagnon J., Kurtz S.K., Liu S.T., Nelson D.F., Hellwege K.-H., Hellwege A.M. (1979). Landolt-Börnstein: Numerical Data and Functional Relationships in Science and Technology, New Series, Group III: Crystal and Solid State Physics, Vol. 11.

[17] Rose J.L. (2014). Ultrasonic Guided Waves in Solid Media.

[18] Auld B.A. (1973). Acoustic Fields and Waves in Solids.

[19] Zhu Z., Li J., Wuf J. (1995). A perturbation analysis of Lamb-wave sensors. Ultrasonics.

[20] Ballantine D.S., White R.M., Martin S.J., Ricco A.J., Zellers E.T., Fye G.C., Wohltjen H. (1997). Acoustic Wave Sensors: Theory, Design & Physico-Chemical Applications.

